# The role of El Niño southern oscillation (ENSO) on variations of monthly *Plasmodium falciparum *malaria cases at the cayenne general hospital, 1996-2009, French Guiana

**DOI:** 10.1186/1475-2875-10-100

**Published:** 2011-04-22

**Authors:** Matthieu Hanf, Antoine Adenis, Mathieu Nacher, Bernard Carme

**Affiliations:** 1 Centre d'Investigation Clinique Epidémiologie Clinique Antilles Guyane CIC-EC CIE 802, Cayenne General Hospital, EHPAD, Avenue des Flamboyants, BP 6006 97300 Cayenne, French Guiana, France; 2 Laboratoire Hospitalo-Universitaire de Parasitologie et Mycologie Médicale, Equipe EA3593, UFR Médecine - Université des Antilles et de la Guyane, Cayenne, Guyane Française, France

## Abstract

**Background:**

Malaria remains a serious problem in French Guiana, which is at potential risk for drought linked with the El Niño Event and where there could be a risk of malaria epidemic after the onset of an El Niño event.

**Methods:**

A time series analysis using ARIMA was developed to investigate temporal correlations between the monthly *Plasmodium falciparum *case numbers and El Niño Southern Oscillation (ENSO) as measured by the Southern Oscillation Index (SOI) at the Cayenne General Hospital between 1996 and 2009.

**Results:**

The data showed a positive influence of El Niño at a lag of three months on *P. falciparum *cases (p < 0.001). The incorporation of SOI data in the ARIMA model reduced the AIC by 4%.

**Conclusions:**

Although there is a statistical link, the predictive value of ENSO to modulate prevention intervention seems marginal in French Guiana. However, additional work should refine the regional dependence of malaria on the ENSO state.

## Background

In French Guiana, malaria remains a serious problem with a number of malaria cases observed every year ranging from 3,500 to 4,500. Both *Plasmodium falciparum *and *Plasmodium vivax *are prevalent. Since 2000, 60% of the cases are due to *P. vivax *[1].

The ENSO phenomenon refers to the cyclic warming and cooling of the equatorial Pacific Ocean coupled with changes in the atmospheric pressure across the Pacific. This is the most important climatic cycle contributing to worldwide interannual variability in climate and the likelihood of climatic anomalies. The two extremes of ENSO are El Niño (a warm event) and La Niña (a cold event), which can create rainfall and temperature fluctuations [2].

The El Niño-Southern Oscillation (ENSO) is the best-known example of quasi-periodic natural climate variability on the interannual time scale. It comprises changes in sea temperature in the Pacific Ocean (El Niño) and changes in atmospheric pressure across the Pacific Basin (the Southern Oscillation), together with resultant effects on world weather. El Niño events occur at intervals of 2-7 years. In certain countries around the Pacific and beyond, El Niño is associated with extreme weather conditions that can cause floods and drought. The other extreme (La Nina) (cooling) is the reverse pattern of climate anomalies.

There is strong evidence that El Niño Southern Oscillation (ENSO) is associated with increased risk of malaria in regions of the world where climate is linked to the ENSO cycle [3]. These include, among others, countries in South America [4-8]. At a regional level, French Guiana is located in a potential risk area for drought linked with the El Niño Event and where there could be a risk of malaria epidemic after the onset of an El Niño event [3].

The purpose of the investigations reported here was to test and quantify the influence of ENSO on malaria transmission in French Guiana using monthly data of malaria cases at the Cayenne General Hospital. Because of the difficulty to discern *P. vivax *malaria relapses from re-infections, this study focused only on *P. falciparum *malaria transmission.

## Methods

### ENSO data

The Southern Oscillation Index (SOI) is designed to measure the strength and phase of the Southern Oscillation. SOI is a measure based on the differences in the atmospheric pressure between Tahiti in the eastern equatorial pacific and Darwin in Australia (West Pacific), expressed as a standard deviation form the norm. SOI is used to quantify the strength of an ENSO event. It is generally negative during an El Niño event and positive during a La Niña event. SOI was taken from the Australian bureau of meteorology [9].

### Malaria data

In spite of some encouraging results to statistically differentiate *P. vivax *malaria relapses from re-infections at individual scale [10], due to the difficulties to discern *P. vivax *malaria relapses from re-infection in endemic areas as French Guiana [11], this study focused only on *P. falciparum *transmission.

All clinical malaria episodes diagnosed for patients consulting the emergency service of Cayenne hospital were retrospectively collected. Malaria was defined as temperature > 38°C at the time of consultation or fever within the past 48 hours associated with Plasmodium asexual forms on a thin blood smear (screening sensitivity: 50 Plasmodium per μl)

Data were extracted from the Parasitological Laboratory files using designated software in accordance with legal procedures concerning confidentiality. Malaria data from 1996 to 2009 were recorded as numbers of cases of *P. falciparum *malaria per month.

### Data analysis

ARIMA models were used to evaluate the relationship between SOI and monthly malaria cases. Before, the ARIMA methodology application, a log-transformation of the series was made to stabilize variance.

Modelling with ARIMA involves the estimation of a series of parameters to account for the inherent dynamics in the time series, including the trends and autoregressive and moving average processes. The general model introduced by Box and Jenkins [12] includes autoregressive and moving average parameters, and explicitly includes differencing in the formulation of the model. An ARIMA (p, d, q) model comprises three types of parameters: the autoregressive parameters (p), number of differencing passes (d), and moving average parameters (q). The multiplicative seasonal ARIMA (p, d, q) (P, D, Q)s model is an extension of the ARIMA method to time series in which a pattern repeats seasonally over time. Analogous to the simple ARIMA parameters, the seasonal parameters are: seasonal autoregressive (P), seasonal differencing (D), and seasonal moving average parameters (Q). The length of the seasonal period is represented by s.

Various permutations of the order (p, d, q) (P, D, Q)s were computed, and the optimal combination of parameters using the « Akaike's information criterion (AIC) » was chosen. The smallest values of Akaike's information criterion (AIC) were set as the standard to identify the best-fit model [12]. The correlogram and partial correlogram graphs were also used to help in fixing the value of orders to include in the model. The likelihood ratio test was used to determine if inclusion of other covariates helped to improve the fit of the model.

In the model, SOI, at lags of 0 to 12 months, respectively, was then fitted into the ARIMA model to screen for potential predictors of the *P. falciparum *case numbers. Lag series significantly associated with malaria, (p-value <0.05), were singled out to fit the best multivariate ARIMA model. The Ljung-Box Q test was applied to ascertain whether the residual series were white noise.

## Results

**Figure 1:**
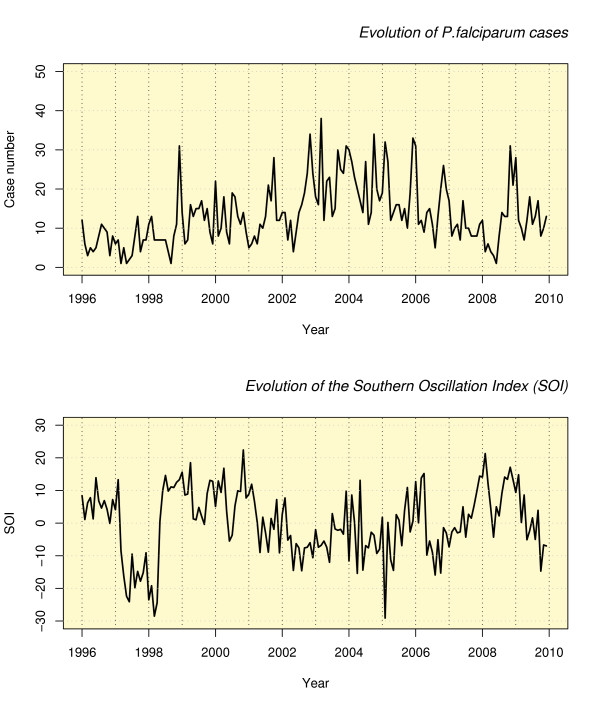
***Plasmodium falciparum *cases at the Cayenne General Hospital plotted with the Southern Oscillation Index (SOI), 1996-2009**.

The monthly observed cases of *P. falciparum *malaria, and SOI, were shown in Figure 1. Of all the models tested, the ARIMA (1, 1, 1) model fitted the data best according to AIC. The best fitting model showed that no significant seasonal pattern explained the monthly cases of *P. falciparum *malaria alone. The results of ARIMA analysis reveal that the logarithm of the malaria cases was significantly influenced by SOI (Table 1).

**Table 1:** Estimated coefficients by ARIMA Model between SOI and logarithmic transformation of malaria cases in Cayenne General Hospital, 1996-2009*

	**Coef**.	**Std. Err**.	P.value
**AR**	0.3813	0.0941	< 0.001

**MA**	-0.8961	0.0479	< 0.001

**SOI lag 3**	-0.0121	0.0049	< 0.001

*

AR: Autoregressive parameter

MA: moving average parameter

AIC of the model: 261.12

In this model, the local moving average parameter is -0.8961 (p =<0.001), and the auto-regression term is 0.3813 (p =<0.001). With SOI data in the model, logarithm of *P. falciparum *cases number was negatively associated with SOI at a lag of three months (β = -0.0121, p = 0.006). The incorporation of SOI data in the ARIMA model reduced the AIC by 4%.

## Discussion

There were two main limitations in the present study. The first one is that this study has used aggregated data coming from the whole heterogenic territory of French Guiana and has to be completed by additional studies at a local scale to determine whether a greater proportion of the variance is predicted, and thus giving a more practical value to the observed correlations in terms of prevention.

The second one is that, although 60% of malaria cases are caused by *P. vivax *in French Guiana, *P. vivax *cases were not included in this analysis due to impossibility to differentiate relapses from re-infections due to missing detailed patient follow-up data.

Nevertheless, this study showed a consistent temporal correlation of ENSO with *P. falciparum *cases in Cayenne General Hospital with a three-month time lag from 1996 through 2009. Although ENSO influence on malaria transmission was suspected in French Guiana, this study is the first to identify a statistically significant correlation between El Niño and malaria cases in this area. A decreasing in SOI three months before was found to act as a positive factor on *P. falciparum *transmission.

The identification of a three-month lag allowed to quantify the delay between ENSO perturbation and consequences on malaria epidemics in French Guiana. Possible explanations for the identified association between ENSO and malaria in French Guiana include the effect of climate on the population dynamics of vectors (mainly *Anopheles darlingi *in French Guiana) through changes in population densities or survival rates, through availability of adequate breeding sites, but also through parasite associated parameters and man-vectors contacts.

The data indicated that ENSO explain 4% of the variation in malaria case numbers in the studied region. At the study scale, the remaining 96% of the variation possibly involved non-climatic causes, such as population immunity and socio-environmental factors that influence the breeding and ecology of mosquito vectors. This result suggested that changes in French Guiana are more likely to be a reflection of the efficiency of local control measures, population movements, therapeutic change, and others social factors [13]. In French Guiana, the role of recent immigration for gold-mining activity is well known as a main drivers of malaria epidemics [14].

To conclude, although there is a statistical link, the predictive value of ENSO to modulate prevention intervention seems marginal. However, the weak predictable consequence of ENSO for malaria transmission is an important piece of information for the development of future early warning systems able to detect efficiently malaria epidemics in the area.

## Competing interests

The authors declare that they have no competing interests.

## Authors' contributions

MH participated in the research design, performed data analysis and interpretation, and prepared the manuscript. AA participated in the interpretation of data and manuscript revision as well. MN provided guidance on data analyses and was involved in the interpretation of data and manuscript revision. BC was responsible for data collection, initiated the study, and was involved in the interpretation of data and manuscript revision. All authors read and approved the final manuscript.
